# Changes in operative and in-hospital outcome of proximal femoral fractures before and after the implementation of work hour restrictions in Switzerland

**DOI:** 10.1007/s00068-026-03204-6

**Published:** 2026-05-13

**Authors:** Alexandra Tewes, Claudio Canal, Othmar Schöb, Valentin Neuhaus

**Affiliations:** 1 University of Zurich, Zurich, Switzerland; 2 Division of Trauma Surgery, Department of Surgery, University Hospital Zurich, Zurich, Switzerland; 3 Department of Surgery, Cantonal Hospital Thurgau, Frauenfeld, Switzerland; 4 Surgical Center, Hirslanden Klinik, Zurich, Switzerland

**Keywords:** Teaching, Working hour regulation, Labor law, Proximal femoral fracture, Patient outcome

## Abstract

**Purpose:**

Surgeon fatigue is a recognized risk factor for patient safety; however, reduced working hours may limit surgical training exposure. In 2005, Switzerland introduced a maximum 50-hour workweek for resident surgeons. In view of current discussions on further reductions (42 + 4 h), concerns persist regarding training quality and workforce capacity. This study evaluates the impact of the 2005 Swiss labor law on patient outcomes, operative characteristics, and intraoperative teaching utilizing a standardized trauma procedure.

**Methods:**

A retrospective analysis of anonymized data from a national database was performed. All patients undergoing operative fixation of trochanteric femur fractures (ICD-10 S72.1) were included. Two four-year periods were compared: pre-regulation (2001–2004) and post-regulation (2016–2019). Primary endpoints were in-hospital complications, mortality, and length of stay. Secondary endpoints included patient characteristics, surgeon seniority, teaching status, operative duration, and time to surgery.

**Results:**

Post-regulation patients were older and exhibited higher American Society of Anesthesiologists classifications despite fewer comorbidities. Complications increased from 9.1% to 17.7%, and mortality from 1.6% to 3.5%. Length of stay decreased from 14 to 9 days. Operative duration decreased by 10 min across all surgeon levels. Resident surgeons independently performed 10% fewer procedures, but teaching operations increased significantly among resident surgeons.

**Conclusion:**

Following the introduction of working hour limitations, supervised teaching increased substantially. Although complication and mortality rates rose—likely reflecting an older and more complex patient population—hospital stays shortened. The proposed 42 + 4-hour model will require targeted compensatory measures to maintain surgical training quality and patient safety.

## Introduction

The weekly working hours of doctors have long been a highly debated topic. In 2003, the United States limited the working hours of medical residents to 80 hours per week and a maximum of 30 consecutive hours a day in an effort to reduce fatigue among medical doctors and, therefore, reduce errors in medical treatment. To this day, a clear causal correlation between the two has not been undoubtedly proven, but there is an increasing number of studies that indicate the negative impact of fatigue on patient outcome [1]. On the contrary, greater clinical experience may lead to a better patient outcome.

In 2004, it was demonstrated that resident doctors with shifts exceeding 24 consecutive hours at the Brigham hospital significantly contributed to an increase in attentional failures and medical errors [2, 3]. Consequently, the Accreditation Council for Graduate Medical Education (ACGME) imposed a limit on consecutive working hours and reduced them to a maximum of 16 hours in 2011; however, these were raised to 28 hours again in 2017 due to staffing shortages and inconclusive results from subsequent studies [4].

In Switzerland, the labor law came into effect on January 1, 2005, applying a maximum limit of 50 working hours per week to all medical residents. One year later, Businger et al. demonstrated that not only was adherence to the 50-hour workweek unattainable but also that the perceived quality of patient care and surgical exposure had declined [5].

Recent surveys conducted among Swiss doctors by the the VSAO (Verband Schweizerischer Assistenz- und Oberärztinnen und -ärzte), the national professional association representing resident and attending physicians in Switzerland, indicate that the maximum working time is still regularly exceeded and the mandatory amount of continuing education is not being adequately provided [6]. Additional surveys reveal that while universities in the country do contribute to the training of personnel, approximately 10% leave the profession due to the high workload and a lack of support in their careers [7]. This significantly exacerbates the shortage of skilled professionals in the health care sector in Switzerland.

In response to concerns regarding physician well-being, patient safety, and recruitment challenges in the Swiss healthcare system, the VSAO proposed the implementation of a 42 + 4-hour workweek. The proposed model includes 42 hours of regular working time dedicated to patient care, along with 4 mandatory hours of structured continuing education. This raises new concerns within the established medical profession regarding the quality of surgical training with less hands-on training and regarding an aggravation of the already substantial staffing shortage.

Given the current relevance of this debate, this study aims to provide an evidence-based assessment of the impact of the 2005 Swiss labor law on surgical training. We hypothesized that the restriction of working hours led to a decrease in resident-performed procedures. Using a standardized traumatological procedure, we further examined temporal trends in patient demographics and in-hospital outcomes before and after the law’s implementation.

## Methods

### Study design and setting

This is a retrospective study conducted using prospective anonymized data from the Working Group for Quality Assurance in Surgery (AQC; Arbeitsgemeinschaft für Qualitätssicherung in der Chirurgie), a voluntary Swiss registry for surgical quality assessment. The AQC was founded in 1995 and has since accumulated data on 2.1 million surgical procedures and patients from 432 Swiss surgical clinics and affiliated doctors interested in improving care quality. Participation in the AQC is voluntary but has been consistently recommended by all presidents of the Swiss Society of Surgery (SGC) since 1999 [8]. Although the AQC was originally developed for surgical departments, traumatological procedures in Switzerland are performed by both general and orthopedic surgeons, as both are accredited for trauma care. While polytrauma management was historically led by general surgeons, current practice has shifted toward interdisciplinary care, with fracture treatment being increasingly performed by orthopedic surgeons [9, 10]. Due to the anonymization of the data, no ethics application was required.

The AQC questionnaire comprises two distinct forms. The first form is designed to capture demographic characteristics, including age and sex. Clinical status is characterized by the American Society of Anesthesiologists’ (ASA) physical status classification and the presence of comorbidities or concomitant injuries. Socioeconomic status is represented by insurance class—that is, private, semi-private, or public. The second form records procedure-related information, such as the interval from hospital admission to surgery; the qualification level of the primary surgeon, such as senior consultant, junior consultant, or resident; and the teaching status of the operation. Operative characteristics include the duration of surgery and the occurrence and severity of perioperative complications, categorized as none, conservatively managed, requiring surgical intervention, resulting in long-term damage, or fatal. Outcome measures encompass length of hospital stay, discharge destination, and in-hospital mortality.

For the period before the introduction of the labor law, the years 2001–2004 were included. For the period after the introduction of this law, we selected a four-year span, encompassing the years 2016–2019, to allow time for the implementation of the legislative changes. The period of the COVID-19 pandemic was deliberately excluded due to the extraordinary strain it imposed on the entire health care system.

We selected a highly standardized traumatological procedure regularly performed by both residents and senior doctors. The study included only patients with a trochanteric femur fracture operatively treated with closed reduction and internal fixation, identified by code 79.15 in the Swiss Classification of Surgical Interventions (CHOP) [11] and diagnosis code S72.1* (ICD-10 - International Statistical Classification of Diseases and Related Health Problems) [12]). The type of implant was not considered in this study.

### Insurance categories

In the Swiss health care system, patients can choose between statutory basic insurance and various supplementary plans. By law, all residents must obtain mandatory basic health insurance. This core coverage can be expanded with semi-private or private supplementary insurance, which provides enhanced benefits—such as broader coverage and additional comforts, including access to single-occupancy hospital rooms. According to prevailing practice, patients with supplementary health insurance are intended to undergo surgery performed by senior consultants, whereas patients only covered by basic insurance may be operated on by a resident surgeon. However, the actual implementation of this arrangement is subject to the discretion of the respective hospital. To date, no statutory framework has been established to govern this practice; rather, it is solely guided by nonbinding recommendations from health insurance providers.

### Teaching

Another element included in the AQC form is the field labelled “Teaching.” This category was defined by the authors as including a less experienced surgeon, usually a resident, performing the surgery under the supervision of a more experienced surgeon, typically a junior or senior consultant. The decision to designate a procedure as a teaching operation lies with the reporting surgeon, as the AQC provides no clear definition or guidance on this classification. This decision is influenced by multiple factors, including the complexity of the surgical intervention, the experience level of the resident in question, the severity of the underlying condition, the patient’s comorbidities, and the individual’s insurance status.

Historically, minor or routine procedures were to a substantial extent performed unsupervised by residents or junior consultants, as demonstrated by data from the United Kingdom. While no comparable data are available for Switzerland, a similar practice can reasonably be assumed. Independently performed procedures were defined by the authors as those conducted by a resident without being labelled as teaching operations [13, 14].

### Endpoints

The primary endpoints of our study were to compare outcomes before and after the introduction of the labour law, focusing on the occurrence of in-hospital complications, mortality, and length of hospital stay.

The secondary endpoints examined changes in the patient cohort (age, sex, comorbidities, ASA-class, insurance status), the classification of the surgeon (senior consultant, junior consultant, resident), the teaching status of the procedure, operation duration, and time to operation.

### Statistical analysis

The data were extracted using the Adjumed Analyze tool (Adjumed Services AG, Zurich, Switzerland) and subsequently analyzed using the Statistical Package for the Social Sciences (SPSS Statistics, Version 24; IBM Corp., Armonk, NY, USA). All statistical analyses were conducted in accordance with conventional standards in biomedical research, with a statistical significance defined as a two-tailed *p*-value < 0.05.

Descriptive statistics were used to summarize patient characteristics, operative variables, and outcomes. Continuous variables (e.g., age, duration of surgery, time to surgery, and length of hospital stay) were presented as means, and differences between the two study periods (2001–2004 vs. 2016–2019) were assessed using the independent samples t-test. Categorical variables (e.g., sex, ASA class, presence of comorbidities, insurance status, surgeon classification, teaching status, occurrence of complications, discharge destination, and in-hospital mortality) were presented as absolute and relative frequencies (%). Comparisons among groups were performed using the Pearson chi-square test; in cases of small, expected cell counts (< 5), Fisher’s exact test was applied.

Where applicable, subgroup analyses were performed to investigate associations between insurance status and surgeon classification (e.g., likelihood of operations being performed by senior consultants among privately insured patients) as well as the relationship between ASA class and intraoperative teaching status.

We utilized backward stepwise binary logistic regression as a statistical tool to explore and identify independent risk factors associated with the likelihood of death and any complication. ASA V patients were excluded as predictors for mortality, since they were always associated with death.

The artificial intelligence software “ChatGPT” (OpenAI, ChatGPT 5 − 0, 2025) and “OpenEvidence” (OpenEvidence Inc., 2025) were partially utilized to check the manuscript for orthographic and grammatical accuracy, to refine the wording, and to assist with the formulation of statistical analyses. No concepts generated by the software were adopted without verification. The authors reviewed and edited the content as needed and take full responsibility for the content of the publication [15, 16].

## Results

### Demographics

The study included 492 patients in the first period and 2424 patients in the second period.

In the cohort prior to the introduction of the labor law, the following demographic characteristics were observed: the mean age was 81.1 years, the gender distribution leaned toward females with 75.2%, 71% presented with relevant comorbidities, and most were classified as ASA II (29.5%) or ASA III (47.6%). Only 19.7% of patients held supplementary insurance.

In the cohort after the introduction of the labor law, patients were significantly older, with a mean age of 82.7 years. The gender distribution was comparable to the other cohort, with 73.9% females. Despite a significantly lower overall comorbidity burden, a higher proportion of individuals presented with an elevated ASA classification, with 47% classified as ASA II and 45.6% classified as ASA III risk-class (Table 1).

**Table 1:** Patient characteristics

Parameter		Total (*n*= 2916)		2001 - 2004 (*n*= 492)		2016 - 2019 (*n*= 2424)		*p* value
		*n*	%	*n*	%	*n*	%	
Age (years)	mean	82.4		81.1		82.7		0.004
Sex	male	754	25.9%	122	24.8%	632	26.1%	0.556
	female	2162	74.1%	370	75.2%	1792	73.9%	
ASA	I (healthy person)	209	7.2%	92	18.7%	117	4.8%	<0.001
	II (mild systemic disease)	1285	44.1%	145	29.5%	1140	47.0%	
	III (severe systemic disease)	1339	45.9%	234	47.6%	1105	45.6%	
	IV (severe systemic disease that is a constant threat to life)	82	2.8%	21	4.3%	61	2.5%	
	V (moribund)	1	0.0%	0	0.0%	1	0.0%	
Comorbidity	yes	1804	62.0%	347	71.0%	1457	60.0%	<0.001
Insurance	statutory	2205	75.6%	395	80.3%	1810	74.7%	0.008
	private	711	24.4%	97	19.7%	614	25.3%	

ASA: American Soceiety of Anesthesiologists classification system

### Complications, mortality, and length of hospital stay

We observed a significant increase in complications after the introduction of the labour law, with 17% after the implementation of the legislation compared to 9% prior to 2005. Similarly, there was a significant rise in mortality, with 3.5% after the introduction of the legislation compared to 1.6% prior to 2005. In binary logistic regression analysis, the treatment period 2016–2019 remained an independent predictor of both complications and mortality when compared to 2001–2004. Additional independent predictors of complications included higher ASA score, presence of comorbidities, increasing age, and longer operative duration. For mortality, male sex and increasing age were additional independent predictors, while ASA score and comorbidities showed trends toward significance (Tables 2, 3 and 4).

However, a significant decrease in length of hospital stay was observed after 2016, with patients staying a median of 9 days in contrast to a median of 14 days prior to 2005 Comment (Table 2).

**Table 2:** In-hospital outcome, teaching and surgeon class

Parameter		Total (*n*= 2916)		2001 - 2004 (*n*= 492)		2016 - 2019 (*n*= 2424)		*p* value
		*n*	%	*n*	%	*n*	%	
Time to surgery (days)	mean	0.9		0.7		1		0.003
Surgeon class	senior consultant	1002	34.4%	145	29.5%	857	35.4%	<0.001
	junior consultant	1129	38.7%	172	35.0%	957	39.5%	
	resident	785	26.9%	175	35.6%	610	25.2%	
Teaching	not specified	557	19.1%	367	74.6%	190	7.8%	<0.001
	no	1395	47.8%	0	0.0%	1395	57.5%	
	yes	964	33.1%	125	25.4%	839	34.6%	
Duration of operation (minutes)	mean	64		74		63		<0.001
Complications	no	2443	83.8%	447	90.9%	1996	82.3%	<0.001
	yes	473	16.2%	45	9.1%	428	17.7%	
Length of stay (days)	mean	10		14		9		<0.001
Discharge	deceased	92	3.2%	8	1.6%	84	3.5%	<0.001
	at home	746	25.6%	142	28.9%	604	24.9%	
	other	357	12.2%	35	7.1%	322	13.3%	
	nursing home	494	16.9%	84	17.1%	410	16.9%	
	old people’s home	444	15.2%	88	17.9%	356	14.7%	
	rehabilitation clinic	783	26.9%	135	27.4%	648	26.7%	
Mortality	alive	2824	96.8%	484	98.4%	2340	96.5%	0.033
	deceased	92	3.2%	8	1.6%	84	3.5%	

**Table 3:** Predictors of mortality

Parameter	Sig.	OR	95% CI	
			LL	UL
2016 - 2019 vs. 2001 - 2004	0.004	5.453	1.7	18
Sex (male vs. female)	0.003	2.208	1.3	3.7
Comorbidities yes	0.072	1.832	0.95	3.5
ASA III and V vs. I and II	0.060	1.671	0.98	2.9
Age (years)	<0.001	1.105	1.1	1.1

CI = confidence intervall, LL = lower limit, OR = odds ratio, Sig. = significance, UL = upper limit

**Table 4:** Predictors of complications

Parameter	Sig.	OR	95% CI	
			LL	UL
2016-2019 vs. 2001-2004	<0.001	2.280	1.6	3.2
ASA III and V vs. I and II	<0.001	1.693	1.3	2.2
Comorbidities yes	0.040	1.353	1.01	1.8
Age (years)	0.002	1.019	1.01	1.03
Duration of operation	<0.001	1.008	1.01	1.01

CI = confidence intervall, LL = lower limit, OR = odds ratio, Sig. = significance, UL = upper limit

### Secondary endpoints

We observed a significant increase in time to surgery following the implementation of the working hours legislation, with a marked difference of approximately seven hours. In contrast, the duration of surgery significantly decreased by approximately 10 min. Further, we found that adjusting for surgeon classification does not attenuate the observed 10-minute reduction, which remains statistically significant across all three categories (Tables 2 and 5).

**Table 5:** Surgeon classification and operation duration based on ASA class and insurance category

Surgeon classification		Senior Consultant				Junior Consultant				Resident			
Time period		2001 - 2004		2016 - 2019		2001 - 2004		2016 - 2019		2001 - 2004		2016 - 2019	
		*n*	%	*n*	%	*n*	%	*n*	%	*n*	%	*n*	%
ASA	I (healthy person)	25	17.2%	47	5.5%	30	17.4%	39	4.1%	37	21.1%	31	5.1%
	II (mild systemic disease)	44	30.3%	371	43.3%	41	23.8%	453	47.3%	60	34.3%	316	51.8%
	III (severe systemic disease)	68	46.9%	416	48.5%	91	52.9%	439	45.9%	75	42.9%	250	41.0%
	IV (severe syst. disease with constant threat to life)	8	5.5%	22	2.6%	10	5.8%	26	2.7%	3	1.7%	13	2.1%
	V (moribund)	0	0.0%	1	0.1%	0	0.0%	0	0.0%	0	0.0%	0	0.0%
Insurance category	statutory	81	55.9%	434	50.6%	153	89.0%	825	86.2%	161	92.0%	551	90.3%
	private	64	44.1%	423	49.4%	19	11.0%	132	13.8%	14	8.0%	59	9.7%
Operation duration (mins)	mean (± SD)	64 (±29)		53 (±35)		79 (±44)		69 (±38)		76 (±29)		66 (±55)	

SD: Standard Deviation, ASA: American Soceiety of Anesthesiologists classification system

There was a marked increase in the proportion of teaching operations. After adjustment for insurance status, there was a reduced operative involvement of junior consultants, 25% fewer procedures for publicly insured patients and 1.3% fewer procedures for privately insured patients, a decrease paralleling the increase among residents, who performed 23.1% more procedures for publicly insured patients and 3% more procedures for privately insured patients (Table 6).

**Table 6:** Percentage of teaching operations based on insurance category for junior consultants and residents

Insurance category	Surgeon classification	2001 - 2004	2016 - 2019
		%	%
Statutory	junior consultant	58.3%	33.5%
	resident	31.0%	54.1%
Private	junior consultant	7.1%	5.8%
	resident	3.6%	6.6%

Further, we found a decrease of 10% in the total number of surgeries performed by resident surgeons since 2016, as indicated by the significant changes in surgeon class. The proportion of operations performed by senior and junior consultants increased by an equal percentage, thereby reflecting a compensatory shift (Table 2).

Considering the significant increase in patients’ ASA classification, a corresponding decrease in the proportion of ASA I patients of similar magnitude as the increase in the proportion of ASA II patients is observed across all three surgeon classes (Table 5).

The insurance status of patients revealed significant changes, with a 5.6% increase in privately insured patients after the implementation of the labor law. Although senior consultants continue to perform the majority of surgeries on patients with supplementary insurance, from 2016 onward, there has been a modest increase in the proportion of these procedures conducted by junior consultants and residents. (Tables 1 and 5)

## Discussion

Using the operative management of proximal femoral fractures as a model procedure, this study evaluated the impact of the introduction of the Swiss labor law in 2005 by examining changes in patient demographics, in-hospital outcomes, and intraoperative teaching practices before and after the law’s implementation.

Despite the differing dataset sizes, the two populations were largely comparable, with a similar sex distribution and only a marginally significant difference of 1.6 years in the mean age. Interestingly, while patient comorbidities appeared to decrease, the proportion of patients classified with higher ASA scores increased. It is important to note that the AQC does not provide a standardized definition of what constitutes a comorbidity, thus leaving the assessment of this patient characteristic to the discretion of the operating surgeon. Nevertheless, we conclude that, overall, although patients present with slightly fewer secondary diagnoses, these conditions appear to be less well-controlled, which likely explains the higher ASA classification.

Given the older and more comorbid patient population, it is unsurprising that we observe a higher complication rate and an increase in mortality in the second group.

The significantly longer duration to operation must be interpreted with caution, as the difference amounts to only 0.3 days, that is approximately 7.2 hours.

Of greater clinical relevance was the significant reduction in hospital length of stay, which decreased by five days on average. The observed 5-day reduction in length of hospital stay is consistent with nationwide Swiss data demonstrating a continuous decline in hospitalization duration over the past two decades [17]. Our findings extend these observations to patients with pertrochanteric proximal femur fractures, confirming that similar efficiency gains have been achieved in this clinical context.

Regarding surgeon seniority, residents now independently perform fewer procedures, with their share decreasing from approximately one-third to one-fourth of all operations. Concurrently, teaching cases among residents increased by over 25%, while teaching involvement among attending surgeons decreased by the same margin. These findings suggest that, following the implementation of the labor law, residents perform a larger proportion of supervised operations, with a corresponding decline in involvement of junior consultants. This trend may be attributed to the increasingly complex and comorbid patient population as well as to rising expectations regarding surgical outcomes and health care standards. Nevertheless, it should be acknowledged that these findings might, at least in part, be attributable to the introduction of the Swiss labour law. We consider it highly unlikely that this is related to the type of implants, as operative times have decreased across all surgeon levels, which supports the assumption of intuitive handling of surgical instruments. Another possible reason for the overall decrease in surgeries performed by residents could be the increase in privately insured patients, who, based on their supplemental hospital insurance (semi-private or private), are contractually entitled to treatment by a board-certified or senior surgeon. This entitlement is anchored in the terms of Swiss supplemental health insurance policies, which typically guarantee free choice of doctor and treatment by a senior (consultant-level) surgeon [18].

This leads to the central research question of this study: Are residents performing fewer operations since the implementation of the revised Swiss labor law? Our data suggest that on average, residents now perform 10% fewer procedures compared to the period before the law came into effect. However, this reduction must be interpreted in context: the patient population has become notably more comorbid and complex, which likely contributes to the shift in operative responsibilities. At the same time, there has been a substantial increase in teaching operations: over 25% more procedures are now performed with direct supervision. Thus, while residents independently perform fewer procedures, they are more frequently integrated into structured teaching environments under senior supervision. This may reflect both the increasing complexity of surgical cases and the rising standards of quality patient care in the Swiss health care system.

### Limitations

The most significant limitation of this study lies in the nature of the dataset. Given the voluntary nature of participation, the absence of a defined registry of participating institutions, and the potential underrepresentation of orthopaedic clinics performing fracture care, a certain degree of selection bias must be assumed. Furthermore, due to complete anonymization, it was not possible to retrospectively trace individual cases. Consequently, it was not possible to perform an external verification regarding the accuracy or consistency of data entries by the operating surgeons. This limited our ability to assess the reliability of specific variables across institutions or over time.

Moreover, the AQC does not provide standardized definitions for several key parameters, such as comorbidities or teaching, over different years. Therefore, the interpretation and documentation of such variables are left to the discretion of the individual surgeon. This lack of uniformity poses a major challenge for data harmonization and limits the comparability of outcomes across cases and surgeon levels. These methodological constraints should be considered when interpreting the results of our analysis.

Over the study period, substantial changes in hip fracture care and hospital organization occurred, including advances in implant design and fixation techniques as well as evolving peri- and postoperative management strategies. These developments represent important potential confounders that may have influenced the observed outcomes.

In addition, this study only captures short-term, in-hospital complications. Post-discharge outcomes and long-term follow-up data are not available, which prevents any conclusions regarding complications or recovery trajectories beyond the hospital stay. This limits the scope of the study to early postoperative outcomes and excludes potential delayed adverse events.

Fourth, the choice of implant was not considered in this analysis. Differences in implant design, quality, or handling characteristics could have a significant impact on surgical outcomes but were not assessed in this study. Therefore, no conclusions can be drawn regarding the influence of implant selection on perioperative results.

Finally, while the study examines the period following the implementation of the Swiss labor law, which introduced a maximum number of weekly working hours, it does not permit any conclusions regarding whether these legal limits were actually observed in clinical practice. Thus, the actual number of hours worked by surgical residents cannot be verified based on the available data.

### Comparison with literature

A systematic review by Hamza et al. revealed no changes in the caseload after the globally introduction of work hour restrictions [19]. In contrast, Muhammad et al. found that duty hour restrictions did not improve patient outcomes, decreased surgical experience for junior residents, and shifted clinical responsibilities to junior consultants [20].

Procedural exposure remains fundamental to surgical training, a discussion that has been particularly prominent in the field of robotic-assisted surgery in recent years. Although robotic surgery is not directly relevant to the traumatological procedure examined in our study, the principles of surgical mastery remain consistent: achieving competence requires adequate case volume, regardless of technique [21]. A study by Grossen et al. suggests that the increasing surgical workforce in Switzerland, combined with reduced working hours and a shift toward outpatient care, is likely to reduce procedural exposure for individual surgeons—highlighting the need to adapt training structures to maintain high standards of education and patient care [22].

Comparable to international findings that reveal the conflicting effects of reduced working hours on surgical training and patient outcomes, a study by Antony et al. indicates that the proposed 42 + 4-hour model in Switzerland will likely require compensatory mechanisms to preserve both educational quality and patient safety [23].

## Conclusion

This study demonstrated that since the introduction of the Swiss labor law, resident surgeons independently perform significantly fewer procedures but are increasingly involved in supervised teaching operations. Although complication rates and in-hospital mortality increased over time, the length of hospital stay markedly decreased, thus reflecting improvements in efficiency and health care delivery. These findings suggest that while working hour regulations may have reshaped surgical training and case allocation, structured supervision would ensure continued educational opportunities while maintaining patient safety.

## Data Availability

The data underlying this study were obtained upon request from the AQC (Arbeitsgemeinschaft für Qualitätssicherung in der Chirurgie). Analyses were performed using the Adjumed Analyze tool. Due to data protection regulations and the terms of data use, the raw data are not publicly available but can be requested from AQC subject to their approval and applicable legal requirements.
